# 2-Deoxy-D-Glucose inhibits aggressive triple-negative breast cancer cells by targeting glycolysis and the cancer stem cell phenotype

**DOI:** 10.1038/s41598-019-39789-9

**Published:** 2019-03-07

**Authors:** Sadhbh O’Neill, Richard K. Porter, Niamh McNamee, Vanesa G. Martinez, Lorraine O’Driscoll

**Affiliations:** 1 School of Pharmacy and Pharmaceutical Sciences & Trinity Biomedical Sciences Institute, Trinity College Dublin, Dublin 2, Ireland; 2 School of Biochemistry and Immunology & Trinity Biomedical Sciences Institute, Trinity College Dublin, Dublin 2, Ireland

## Abstract

Due to limited availability of pharmacological therapies, triple-negative breast cancer (TNBC) is the subtype with worst outcome. We hypothesised that 2-Deoxy-D-Glucose (2-DG), a glucose analogue, may hold potential as a therapy for particularly aggressive TNBC. We investigated 2-DG’s effects on TNBC cell line variants, Hs578T parental cells and their isogenic more aggressive Hs578Ts(i)_8_ variant, using migration, invasion and *anoikis* assays. We assessed their bioenergetics by Seahorse. We evaluated metabolic alterations using a Seahorse XF Analyzer, citrate synthase assay, immunoblotting and flow cytometry. We assessed the cancer stem cell (CSC) phenotype of the variants and 2-DG’s effects on CSCs. 2-DG significantly inhibited migration and invasion of Hs578Ts(i)_8_ versus Hs578T and significantly decreased their ability to resist *anoikis*. Investigating 2-DG’s preferential inhibitory effect on the more aggressive cells, we found Hs578Ts(i)_8_ also had significantly decreased oxidative phosphorylation and increased glycolysis compared to Hs578T. This is likely due to mitochondrial dysfunction in Hs578Ts(i)_8_, shown by their significantly decreased mitochondrial membrane potential. Furthermore, Hs578Ts(i)_8_ had a significantly increased proportion of cells with CSC phenotype, which was significantly decreased by 2-DG. 2-DG may have benefit as a therapy for TNBC with a particularly aggressive phenotype, by targeting increased glycolysis. Studies of more cell lines and patients’ specimens are warranted.

## Introduction

Triple negative breast cancer (TNBC), a collective term for invasive breast cancers that lack expression of estrogen receptor, progesterone receptor and HER2, is responsible for 15–20% of breast cancers and it accounts for a disproportionate number of breast cancer deaths. Poor outcome corresponds with the innate aggressiveness of many TNBC tumours, augmented by the lack of targeted treatments. Based on the phenotype, TNBCs do not respond to endocrine or other currently available targeted therapies, such as trastuzumab (Herceptin), which has had great success in the treatment of HER2-overexpressing breast cancers. While conventional chemotherapy (*e.g*. platinum-containing drugs) regimens have proven beneficial for early stage TNBC, more advanced aggressive TNBC disease typically responds poorly and progresses rapidly, thus the overall outcome is poor^1^. New therapies are therefore an urgent unmet medical need for the treatment of advanced TNBC.

Cancer cells have different bioenergetic requirements than non-malignant cells *i.e*. because they often grow at faster rates, they not only need to generate energy, but also building blocks to support cell proliferation. This means that they usually switch their usual source of energy, oxidative phosphorylation (carried out in the mitochondria) to glycolysis, which is less efficient for generating ATP but results in the production of intermediaries that can then be incorporated into biosynthetic pathways^2^. Thus, cultured cancer cells tend to have increased glycolytic flux and increased lactic acid production and efflux, even under aerobic conditions; a phenomenon known as the Warburg effect.

Recent studies have shown a link between aberrant, mostly glycolytic metabolism and cell migration and invasion (reviewed in^3^). Enhanced glycolysis results in increased lactate secretion and a decrease in extracellular pH, events that promote normal, non-cancerous cell death and enhanced protease-mediated degradation of the extracellular matrix (ECM). Furthermore, several enzymes involved in glycolysis promote epithelial-to-mesenchymal transition (EMT), which is associated with the acquisition of migratory and invasive capacities in cancer cells^4,5^.

2-Deoxy-D-glucose (2-DG) is a synthetic analogue of glucose in which the C-2-hydroxyl group has been replaced by hydrogen. Due to this modification, 2-DG competes with glucose for uptake into cells via the GLUT facilitative transporters but, because it cannot be metabolised for energy, it is cytotoxic to the cells^6^. As cancer cells exhibit higher rates of glycolysis for energy compared to normal cells, the effects of 2-DG should be more selective for cancer cells and should effectively starve cancer cells of energy. Due to this, a limited number of clinical trials have been undertaken with 2-DG. Two phase I dose-escalating trials of 2-DG as a single agent or in combination with docetaxel, in patients with advanced solid tumours, successfully identified clinically-useable doses with tolerable side-effects^7,8^.

In this study, we use a unique isogenic TNBC cell line pair *i.e*. Hs578T and its more invasive subclone variant Hs578Ts(i)_8_^9–11^. Given the established links between aberrant metabolism and increased migration and invasion, we hypothesised that our more aggressive cell variant would display metabolic alterations that would make it more susceptible to the effects of 2-DG. Here we show that enhanced migratory ability correlates with metabolic dysfunction, and that treatment with 2-DG decreases migration, invasion and *anoikis* in our model of aggressive TNBC.

## Methods

### Cell culture

Hs578T (ATCC, Manassas, VA, USA) and its isogenic sub-clone Hs578Ts(i)_8_ (a gift from Dr. Linda Hughes and Dr. Susan McDonnell)^9^ were cultured in Dulbecco’s modified Eagle’s medium (DMEM) (Sigma–Aldrich, St. Louis, USA) supplemented with 10% foetal calf serum (FCS) (Biosciences, Co. Dublin, Ireland), 2 mM L-glutamine (Sigma–Aldrich) and 10 µg/ml insulin (Sigma–Aldrich), constituting complete medium, at 37 °C and 5% CO_2_. The Hs578Ts(i)_8_ isogenic variant has been reported to have significantly increased capacity to proliferate, migrate, invade through ECM and generate tumours in mice^9^.

### Migration assay

Hs578T and Hs578Ts(i)_8_ variants were seeded at 1 × 10^5^ cells/well in a 24-well plate (COSTAR, Corning, New York, USA), allowed to attach overnight and grown to confluency. Cell monolayers were scratched with a 200 µL pipette tip and washed 3 times with complete medium. To assess the influence of 2-DG on migration, 500 µL of medium with 1% FCS and containing 15 mM* 2-DG (Sigma-Aldrich) or 500 µL of medium containing 1% FCS only as control was then added to appropriate wells (Sigma-Aldrich). The wounded areas were monitored by phase contrast microscopy and migration was quantified using NIH Image J Software 24 hr after treatment. [*Of note: a series of complementary experiments were performed using 600 micro-molar, 2-DG; see Supplemental Fig. 4].

### Invasion assay

Invasion assays were performed using 8 µm pore size 24-well transwell chambers (BD Biosciences, Dun Laoghaire, Co. Dublin, Ireland). Chambers were coated with ECM (Sigma-Aldrich) as we previously described^12^. Hs578T and Hs578Ts(i)_8_ variants (5 × 10^4^ cells/chamber) re-suspended in medium with 1% FCS were then seeded in the chamber and allowed to attach overnight. 2-DG (final concentration 15 mM) or medium containing 1% FCS alone as control was added. 400 µL of medium containing 10% FCS was added to the lower compartment of the 24-well plate to create a serum gradient. Cells were allowed to migrate for 24 hr. After this period, cells in the chamber were removed using a PBS-soaked Q-tip and migrated cells were stained with 1% crystal violet (Sigma-Aldrich) prepared in PBS. Images were taken using a phase contrast microscope and crystal violet was subsequently solubilised in 10% acetic acid (Sigma-Aldrich), and absorbance was measured at 595 nm on a FluorStar OPTIMA plate reader (BMG Labtech, Ortenburg, Germany).

### *Anoikis* assay

Most breast cancers are of epithelial cells. Epithelial cells typically do not exist in suspension but are attached to a basement membrane. For such cells to survive in suspension, as required for circulating tumour cells to be transported in the blood stream or lymphatics and progress to forming tumour metastasis, the cells must evade a form of apoptosis termed *anoikis*. We mimic this situation *in vitro* by coating tissue culture plates with Poly(hydroxyethyl methacrylic) acid (p-HEMA; Sigma-Aldrich) and thus inhibiting the ability of the cells to attach to the tissue culture plastic. We subsequently assessed the ability of the cells to survive i.e. to resist *anoikis*. For this, 24-well plates were coated with 200 µL p-HEMA as we previously described^13^. Hs578T and Hs578Ts(i)_8_ cell variants were then seeded at 1 × 10^5^ cells/well and allowed to attach overnight. For those cells to be treated with 2-DG, a final concentration of 15 mM was used; complete medium was used as control. After 24 hrs, 50 µL of Alamar blue dye (Invitrogen, Carlsbad, California, United States) was added to each well and incubated again at 37 °C/5%CO_2_ for 3.5 hr. Absorbance was measured at 570 nm, with a reference wavelength of 600 nm using a FluorStar OPTIMA plate reader.

### Extracellular flux analysis of bioenergetics parameters

The oxidative phosphorylation and glycolytic rates of Hs578T and Hs578Ts(i)_8_ cell variants were assessed using a Seahorse XF^e^24 Analyzer (Agilent Technologies Inc., Santa Clara, CA, US). Assays were performed according to manufacturers’ instructions. Briefly, cells were seeded at 1.8 × 10^4^ cells/well in a 24-well XF microplate (Seahorse Biosciences) and allowed to attach overnight. Cells were washed with glucose-free seahorse XF assay medium (Agilent Technologies Inc.) supplemented with 10 mM D-(+)-glucose (Sigma-Aldrich) and 525 µL of glucose-free seahorse XF assay medium (Agilent Technologies Inc.) supplemented with 10 mM D-(+)-glucose was added to each well. Specific inhibitors and uncouplers were prepared in XF assay media supplemented with 10 mM D-(+)-glucose for sequential addition at the appropriate final concentrations of oligomycin A as 1 µg/ml, FCCP as 300 µM, rotenone as 1 µM, antimycin A as 1 µM, and 2-DG as 30 mM (all Sigma-Aldrich). Following completion of the run, the cells were lysed in the well for protein isolation and quantification. Oxygen consumption rate (OCR) and extracellular acidification rate (ECAR) readings were normalised using these protein concentrations.

### Citrate synthase activity analysis

Hs578T and Hs578Ts(i)_8_ cell variants were seeded at 1 × 10^5^ cells/T25 flasks and incubated for 24 hrs. Cells were collected by scraping and centrifuged at 133 g for 5 min, washed in 1 ml PBS and centrifuged again. Cell pellets were re-suspended in 60 µL PBS and underwent 3 freeze-thaw cycles in liquid nitrogen to lyse cells. Protein was quantified using the BCA assay (Bio-Rad Laboratories, Hercules, California, United States). Citrate synthase (CS), a measure of mitochondrial abundance, was determined spectrophotometrically at 30 °C. Aliquots (10 µL) of the diluted cellular homogenates were used for the assay. Samples were prepared in triplicate by combining the following reagents: 800 µL 0.2 mM Tris (pH 8.1), 30 µL 0.3 mM acetyl-CoA, 100 µL 0.1 mM of (5,5-dithio-bis-(2-nitrobenzoic acid, DTNB) (all reagents were from Sigma-Aldrich). Reagents and cell lysates were placed in 1 ml quartz cuvettes and inserted into a Libra S12 spectrophotometer (Biochrom, Cambourne, United Kingdom) maintained at 30 °C. Baseline readings were taken at 412 nm for 2 min using a flatbed recorder (Kipp and Zonen, Delft, Netherlands) set at 10 mm/min, 0.2 V. 0.5 mM oxaloacetate (Sigma-Aldrich) was added and the absorbance was read for a further 2 min. CS activity was subsequently calculated from the graph recorded using the flatbed recorder according to the following formula: nmol/min^−1^ mg^−1^ protein = (Δ absorbance/min × 1000 × 1)/((13.6 mM^−1^ cm^−1^ × 10 µL) × (Protein concentration mg/mL^−1^))^14^.

### Voltage-dependent anion channel 1 expression analysis by immunoblotting

As another means to measure relative mitochondrial abundance, we assessed voltage-dependent anion channel 1 (VDAC) expression. Total cellular proteins (50 µg) were resolved on NuPage® Novex® 4–12% Bis-Tris SDS-PAGE (Biosciences) and transferred to polyvinylidene difluoride membrane (Millipore, Billerica, Massachusetts, USA). Membranes were incubated overnight with primary antibodies including VDAC (Abcam, Cambridge, United Kingdom, 1:1000 dilution)^15^ and β-actin (Sigma-Aldrich, 1:1000 dilution)^16^ and for 1 hr with horseradish peroxidase-conjugated secondary antibodies anti-rabbit (Cell Signaling, 1:1000 dilution) and anti-mouse (Cell Signaling, Danvers, Massachusetts, USA, 1:1000 dilution), respectively. Proteins were visualised by chemiluminescence (Millipore). Detection was performed with a Chemidoc exposure system (Bio-Rad Laboratories).

### Membrane potential analysis by flow cytometry

For mitochondrial membrane potential analysis, Hs578T and Hs578Ts(i)_8_ cell variants were seeded at 1 × 10^5^ cells/well in a 6-well plate (CoStar) and allowed to attach overnight. Cells were then trypsinised, washed with PBS, re-suspended and incubated in 1 ml 10 µM 10-n-Nonyl Acridine Orange (NAO, Molecular Probes, Eugene, Oregon, United States) in 5% FCS in PBS for 10 min in the dark. Unstained cells were incubated in 5% FCS in PBS for 10 min. Cells were subsequently centrifuged at 164 g for 5 min at room temperature, washed twice in PBS, re-suspended in 200 µL PBS and transferred to flow cytometry tubes. Cell staining for NAO was measured using a FACSCanto II flow cytometer (BD Biosciences, Franklin Lakes, New Jersey, USA) and analysed using BD FACSDIVA software.

### Inhibition of Pyruvate Dehydrogenase Kinase using Dichloroacetate

#### Cytotoxicity Assay

Hs578T and Hs578Ts(i)_8_ cell variants were seeded at 5 × 10^4^ cells/well in a 24-well plate and allowed to attach overnight. Cells were then treated with 0, 5, 10 and 20 mM dichloroacetate (DCA, Sigma-Aldrich) for 24 hr. Proliferation was measured using the acid phosphatase assay as previously described^17^.

#### Seahorse Extracellular Flux Analysis

Seahorse extracellular flux analysis was performed as described in *Extracellular flux analysis of bioenergetics parameters* except that, following their seeding and attachment Hs578Ts(i)_8_ cells were treated with 5 mM DCA for 24 hr. Seahorse extracellular flux analysis proceeded as before.

### Cancer stem cell phenotype analysis by flow cytometry

The expression of CD44 and absence of CD24 (CD44^+^/CD24^−^) is characteristic of breast CSCs. To evaluate these, Hs578T and Hs578Ts(i)_8_ cell variants were seeded at 1 × 10^5^ cells in a 6-well plate and allowed to attach overnight. They were subsequently trypsinised, blocked with 10% FCS in PBS and stained with APC-conjugated anti-CD24 (1:100) (eBioscience, San Diego, California, USA) and FITC-conjugated anti-CD44 (1:400) (eBioscience) for 30 min at 4 °C. Staining was assessed in a FACSCanto II flow cytometer, followed by analysis using BD FACSDiva software. To assess the effects of 2-DG on the CSC population Hs578T and Hs578Ts(i)_8_ cell variants were seeded at 1 × 10^5^ cells in a 6-well plate and allowed to attach overnight. Cells were treated with 2-DG (final concentration 15 mM) for 24 hours. They were subsequently trypsinised, blocked with 10% FCS in PBS and stained with APC-conjugated anti-CD24 (1:100) (eBioscience, San Diego, California, USA) and FITC-conjugated anti-CD44 (1:400) (eBioscience) for 30 min at 4 °C. Staining was assessed in a FACSCanto II flow cytometer, followed by analysis using BD FACSDiva software.

### Statistical analysis

Student’s unpaired t-test was used to compare data generated from Hs578T and Hs578Ts(i)_8_ cell variants. Statistical analysis was performed using GraphPad Priam 5 (GraphPad Software, Inc., San Diego, CA, USA). Results were expressed as a mean of a minimum of three independent experiments ± SEM. Statistical significance was set at *p < 0.05, **p < 0.01, ***p < 0.001.

## Results

### 2-DG inhibits migration, invasion and resistance to anoikis in an aggressive TNBC cell line variant

**Figure 1:**
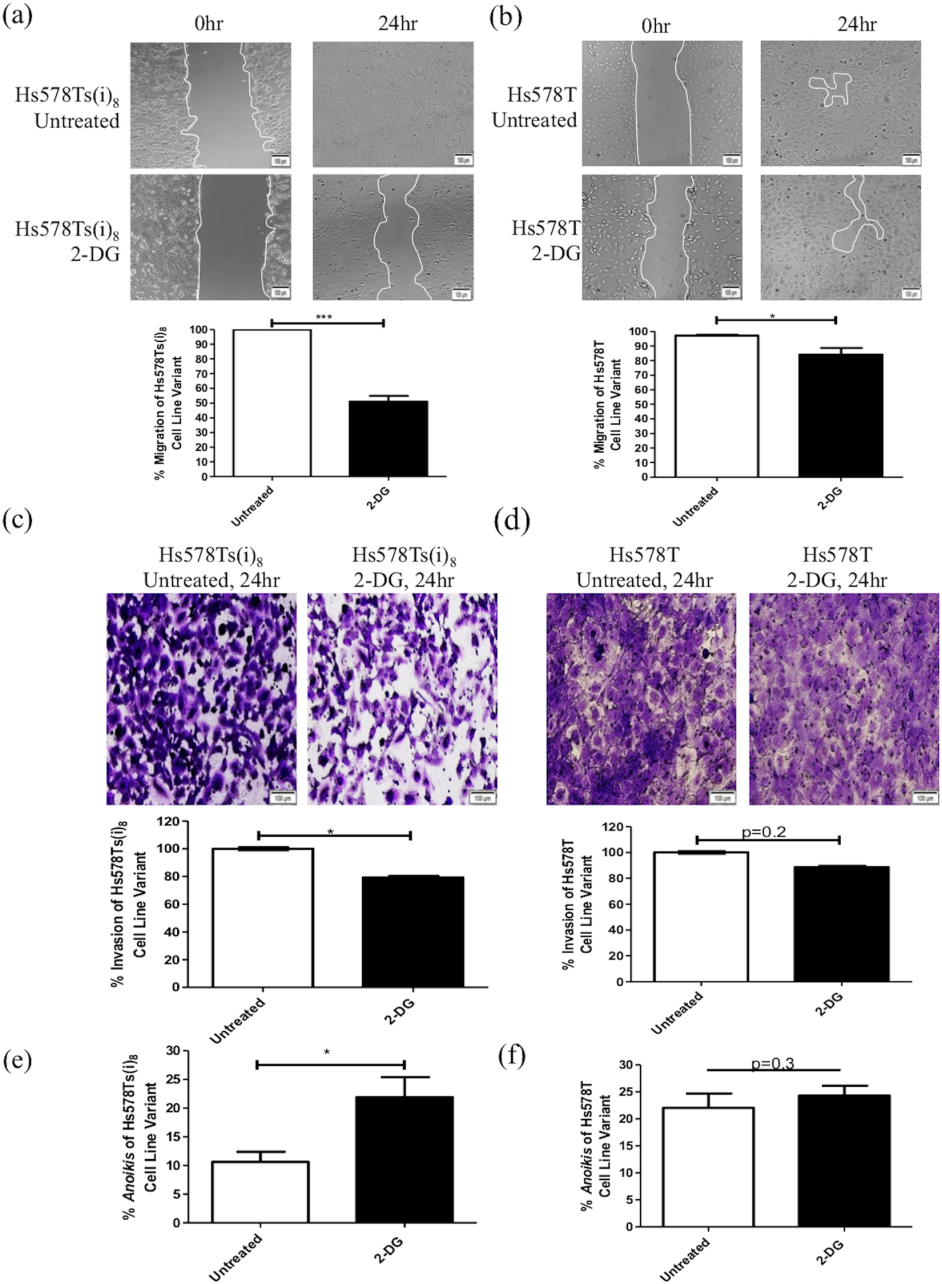
2-Deoxy-D-glucose significantly decreases the migration, invasion and resistance to *anoikis* of Hs578Ts(i)_8_ compared to Hs578T cells. (**a**,**b**) Wound-healing assays indicate that 2-DG significantly decreases the rate of migration of the Hs578T(i)_8_ and Hs578T cell variants, with effects on the Hs578T(i)_8_ population being most substantial. (**c**,**d**) Invasion assays indicate 2-DG significantly decreases the rate of invasion of Hs578T(i)_8_, but not Hs578T, cells; (**e**,**f**) *anoikis* assays showed 2-DG to significantly decreases the apoptosis resistance of the Hs578T(i)_8_ variant, but not the Hs578T cells. Data is expressed as the mean ± SEM of n = 3 experiments, where *p < 0.05, **p < 0.01, ***p < 0.001.

Prior to progressing to more extensive analyses, our initial study was to confirm that the Hs578Ts(i)_8_ cell variant compared to the Hs578T population had a significantly (p = 0.002) greater ability to migrate (Supplemental Fig. 1a), invade through ECM (p = 0.0002) (Supplemental Fig. 1b) and evade death through the form of apoptosis known as *anoikis* (p = 0.03) (Supplemental Fig. 1c). This confirmed that the Hs578Ts(i)_8_ cell variant has a more aggressive phenotype than its isogenic parent cell line Hs578T. Subsequently investigating, initially by wound-healing assay, if 2-DG could influence cellular migration we observed that the presence of 2-DG resulted in a significant decrease in the ‘wound’ closure of both Hs578Ts(i)_8_ (by 49%, p = 1.4 × 10^−06^; Fig. 1(a)) and Hs578T (by 13%, p = 0.03; Fig. 1b) cells; *albeit* the decrease in migration of Hs578Ts(i)_8_ cells was substantially greater than that observed for Hs578T cells. In relation to invasion through ECM, 2-DG significantly (p = 0.02) decreased the rate of Hs578T(i)_8_ cell invasion (Fig. 1c), but not (p = 0.14) that of Hs578T cells (Fig. 1d). Furthermore, 2-DG significantly (p = 0.02) decreased the ability of the Hs578Ts(i)_8_ cells to resist *anoikis* (Fig. 1e), although *anoikis* of Hs578T cells was not significantly (p = 0.32) affected (Fig. 1f).

### Hs578Ts(i)_8_ cells have altered bioenergetics compared to Hs578T cells

**Figure 2:**
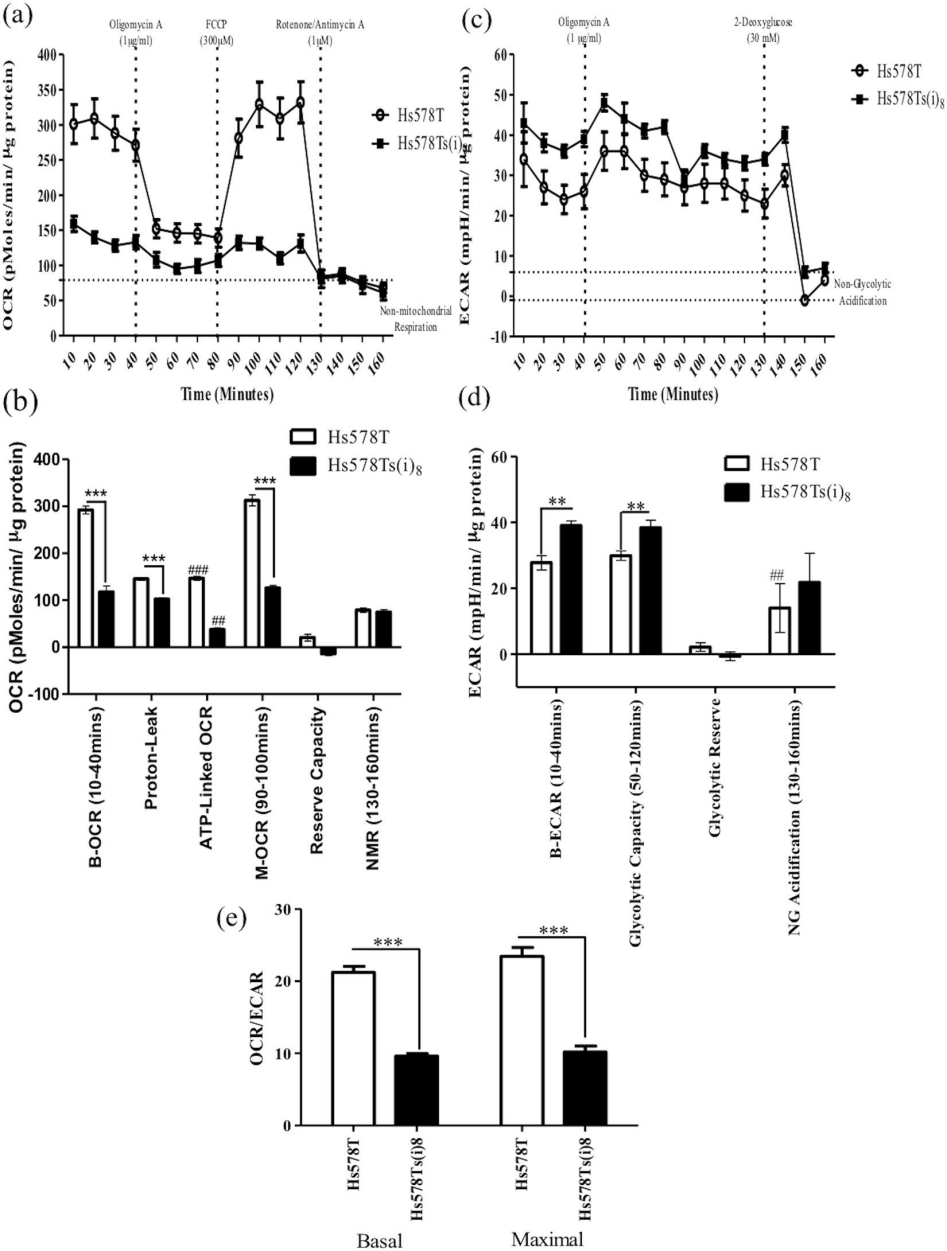
Bioenergetics of the Hs578T and Hs578Ts(i)_8_ cell variants. (**a,b**) There is a significant decrease in the rate of basal oxygen consumption (OCR) in the Hs578Ts(i)_8_ cells compared to the Hs578T cells. Addition of oligomycin A resulted in a significant decrease in the OCR of the Hs578Ts(i)_8_ cells and thus a significant difference in the OCR between the Hs578Ts(i)_8_ and Hs578T variants. Following addition of FCCP, OCR significantly increased in the Hs578Ts(i)_8_ cells. Finally, upon addition of rotenone, oxygen consumption was decreased substantially, a significant decrease from Hs578Ts(i)_8_ cells’ maximal OCR. (**c**,**d**) A significant increase in the basal rate of glycolysis was observed in the Hs578Ts(i)_8_ cells compared to the Hs578T cells. Following oligomycin A addition, the ECAR increased significantly in the Hs578Ts(i)_8_ cells compared to the Hs578T cells. Finally, following addition of 2-DG the ECAR decreased substantially in the Hs578Ts(i)_8_ and Hs578T cells. (**e**) The ratio of the basal and maximal OCR and ECAR is shown, this indicates that the OCR/ECAR ratios were significantly decreased in the Hs578Ts(i)_8_ cells compared to the parental Hs578T cells. Data is expressed as the mean ± SEM of n = 3 experiments, where **/^##^p < 0.01, ***/^###^p < 0.001, *Hs578Ts(i)_8_ vs Hs578T vs, ^#^Hs578T vs Hs578T.

As 2-DG is known to impair cell glycolysis, we investigated whether the mechanism behind decreased migration, invasion and resistance to *anoikis* in the Hs578Ts(i)_8_ cell variant, in response to 2-DG (typically not observed with the Hs578T cells), may be related to altered metabolism in the more aggressive Hs578Ts(i)_8_ population. To evaluate this, metabolic parameters were analysed in both cell line variants using a Seahorse Extracellular Flux Analyser. Basal OCR (mitochondrial plus non-mitochondrial oxygen consumption) were measured and Hs578T(i)_8_ cells were found to have significantly (2.1-fold, p = 7.6 × 10^−6^) lower OCR than their parental Hs578T cells (Fig. 2a,b). Following addition of the ATPase inhibitor, oligomycin A, the OCR was significantly decreased in both cell line variants; however, the decrease in the OCR of the Hs578Ts(i)_8_ variant (37.8 pMole/min/µg protein, p = 0.084) was substantially smaller than for Hs578T cell line variant (146.8 pMole/min/µg protein, p = 0.00019) (Fig. 2a,b). An uncoupler of oxygen consumption from ATP synthesis, FCCP, was subsequently added to give a measure of the maximal OCR of the cell. This indicated a significantly (2.5-fold, p = 6.8 × 10^−6^) decreased maximal OCR for the Hs578Ts(i)_8_ variant compared to the parental Hs578T variant. Of note, the maximal OCR for both cell line variants were not greater than their basal OCR, suggesting both variants are working at their maximal capacity under basal conditions (Fig. 2a,b). Subsequent additions of rotenone and antimycin A completely inhibited oxidative phosphorylation by targeting complex I and complex III, respectively, so that the OCR recorded following addition of rotenone/antimycin A was due to non-mitochondrial oxidative phosphorylation. No differences were observed between the two cell variants in the non-mitochondrial oxidative phosphorylation (Fig. 2a,b). This data demonstrates that Hs578Ts(i)_8_ cells have a significantly (p = 7.6 × 10^−6^) decreased ability to undergo oxidative phosphorylation. This can be seen in the basal OCR rates, which is 292 pMoles/min/µg protein in Hs578T cells, but only 75 pMoles/min/µg protein in the Hs578Ts(i)_8_ cells.

As we hypothesised, the decrease in OCR in Hs578Ts(i)_8_ cells was accompanied by a concomitant increase in glycolysis as shown by the ECAR; this is a phenomenon known as the Warburg effect (Fig. 2a,b). Specifically, there was a significant (1.5-fold, p = 0.005) increase in the rate of basal glycolysis of the Hs578Ts(i)_8_ variant compared to that of the parental Hs578T cells. There was also a significant (1.3-fold, p = 0.01) increase in another ECAR parameter, *i.e*. glycolytic capacity, which is a measure of maximal glycolysis (Fig. 2c,d). As was observed with oxidative phosphorylation (Fig. 1a,b), Hs578Ts(i)_8_ and Hs578T cells appear to be working at their maximal glycolytic capacity, as there was no difference between the basal ECAR and maximal ECAR rates (Fig. 2c,d). Due to these bioenergetic changes, the basal and maximal OCR/ECAR ratios were significantly decreased (2.2-fold, p = 1.5 × 10^−5^ and 2.3-fold, p = 0.001, respectively) in the Hs578Ts(i)_8_ cell variant compared to the parental Hs578T cells. This is likely due to the decrease in the OCR and the increase in ECAR observed in the Hs578Ts(i)_8_ cell line variant (Fig. 2e).

### Decreased OCR in Hs578Ts(i)_8_ cells is not due to reduced mitochondrial biomass

**Figure 3:**
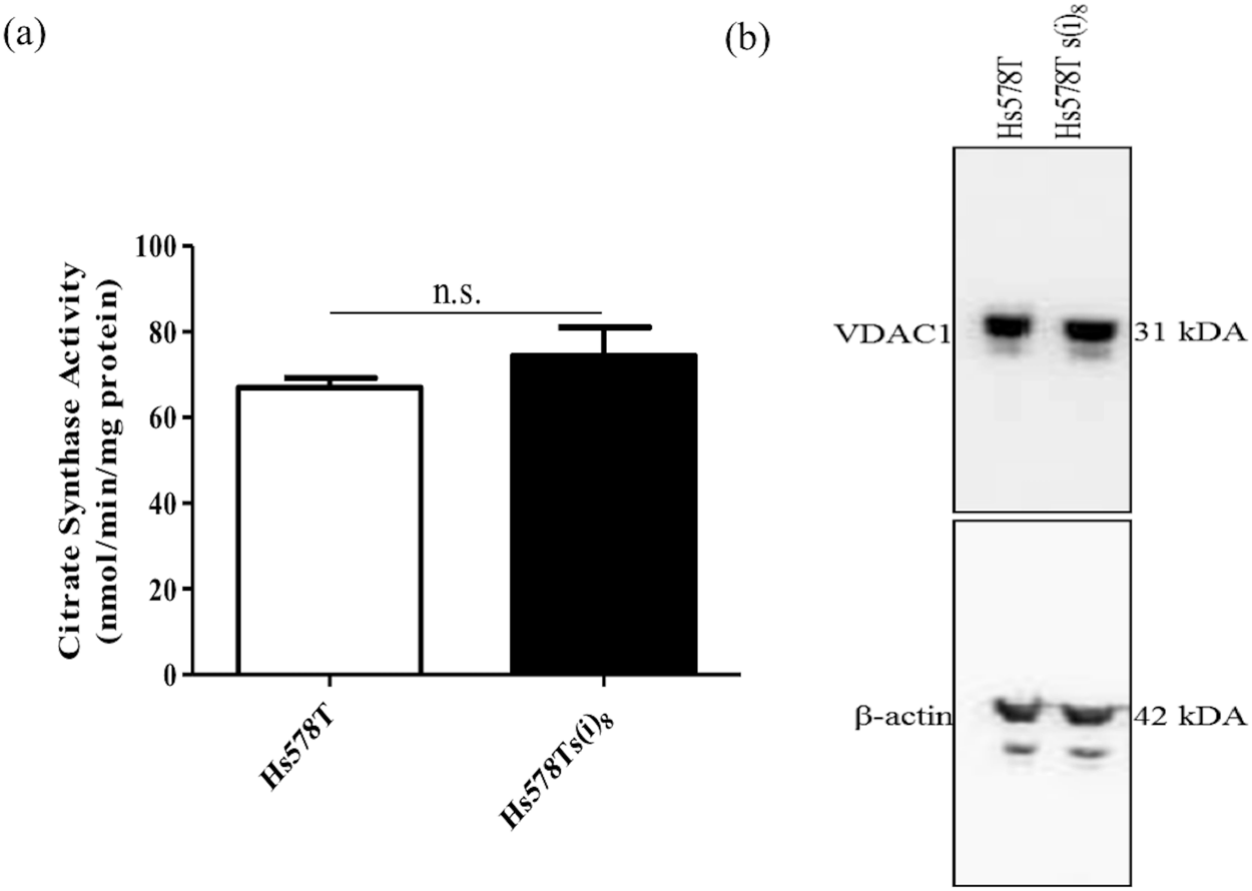
Evaluation of potential alterations in the mitochondrial biomass. Analysis of mitochondrial biomass by **(a)**. The citrate synthase assay and **(b)**. Immunoblot for VDAC1 showed no significant differences between Hs578T and Hs578Ts(i)_8_ cell variants. Data is expressed as the mean ± SEM of n = 3 experiments, differences were not statistically significant (n.s.). Note: the immunoblot was cropped to approximately 6 gel bandwidths of the relevant band. VDAC1 was probed for, the transmembrane was stripped and re-probed with the anti-β-actin antibody. Due to the large qualities of VDAC1 in the cell lysates, complete stripping did not occur and faint VDAC1 bands are still visible when presenting β-actin, due to the large quantities of VDAC1. However, their size differences make them clearly distinguishable.

To further investigate whether the significant decrease in oxidative phosphorylation observed in the Hs578Ts(i)_8_ cell variant compared to the Hs578T cells is due to a decrease in mitochondrial biomass, citrate synthase (CS) activity was then measured. CS is the enzyme that catalyses the first step of the Krebs cycle where it converts acetate from acetyl CoA and oxaloacetate into citrate; this enzyme is localised in the mitochondrial matrix in virtually all cell types and it is a quantitative enzyme marker for intact mitochondria. As shown in Fig. 3a, however, Hs578T and Hs578Ts(i)_8_ variants have similar CS activity. To confirm these results, we investigated the expression of VDAC1. VDAC1 is a major calcium ion transport channel and is considered the most abundant outer mitochondrial matrix protein. Immunoblotting showed similar levels of VDAC1 expression in both Hs578T and Hs578Ts(i)_8_ suggesting, again, that mitochondrial biomass is not significantly different among these two variants (Fig. 3b; See Supplemental Fig. 2 for triplicate repeats and uncropped gels). Together, these results suggest that the decrease in oxidative phosphorylation observed in Hs578Ts(i)_8_ compared to Hs578T is not due to reduced mitochondrial biomass.

### Pyruvate dehydrogenase kinase activity is not increased in the Hs578Ts(i)_8_ cell line variant

**Figure 4:**
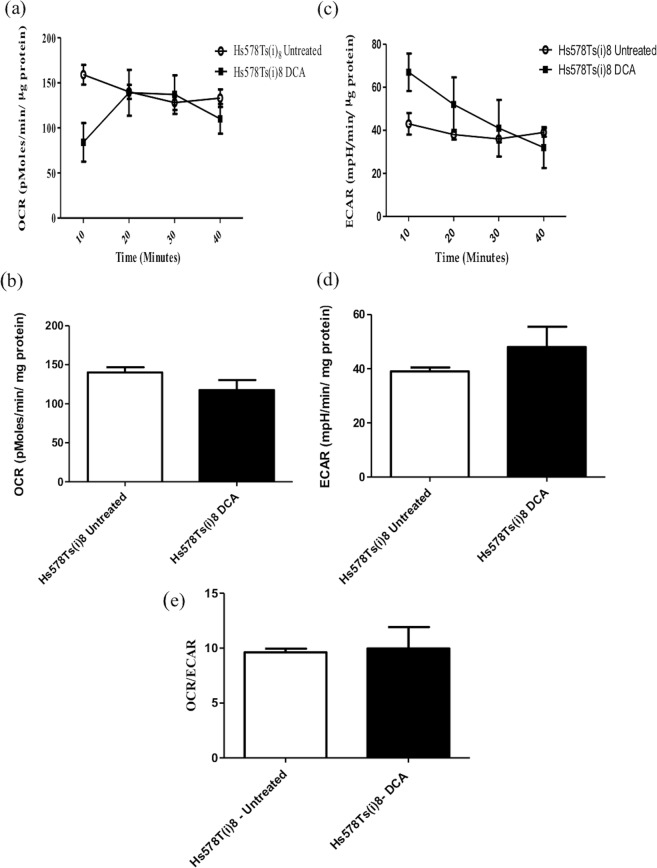
Bioenergetics of the Hs578T and Hs578Ts(i)_8_ variants following DCA treatment. (**a**,**b)** Following DCA treatment the basal OCR of the Hs578Ts(i)_8_ cells did not change significantly from the OCR observed without treatment. (**c**,**d**) Similarly the ECAR does not significantly change following treatment with DCA. (**e**) The ratio of the OCR and ECAR for the untreated and DCA treated Hs578Ts(i)_8_ cells is similar, showing that PDK is not implicated in the switch of the Hs578Ts(i)_8_ cells from oxidative phosphorylation to glycolysis. Data is expressed as the mean ± SEM of n = 3 experiments.

One of the possible causes of metabolic alteration in cancer cells is a decreased substrate supply and this can sometimes be explained by increased pyruvate dehydrogenase kinase (PDK) activity. PDK is an inhibitor of pyruvate dehydrogenase (PD) which converts pyruvate produced during glycolysis into acetyl CoA. The resulting acetyl CoA enters the Krebs cycle and provides reducing equivalents for oxidative phosphorylation. When PDK is increased the supply of acetyl CoA to the Krebs cycle is restricted, resulting in increased glycolysis^18^. Increased PDK expression has been detected in many cancers and, therefore, its inhibitor dichloroacetic acid (DCA) has been studied as a possible anti-cancer treatment^19,20^. To investigate whether PDK expression is increased in the Hs578Ts(i)_8_ cell line variant thus reducing the supply of acetyl CoA to the mitochondria for oxidative phosphorylation, Hs578Ts(i)_8_ cells were treated with 5 mM DCA for 24 hrs prior to analysis with the Seahorse Extracellular Flux analyser; this concentration was chosen because it did not affect cell viability (Supplemental Fig. 2). We observed that DCA inhibition of PDK did not increase oxidative phosphorylation in these Hs578Ts(i)_8_ cells (Fig. 4a,b), suggesting PDK is not involved in the observed switch to glycolysis in these cells. Similarly, DCA treatment did not affect glycolysis in Hs578Ts(i)_8_ cells, as deduced from ECAR rates (Fig. 4c,d). As thus expected, the OCR/ECAR ratios were similar before and after DCA treatment (Fig. 4e).

### Mitochondria are dysfunctional in Hs578Ts(i)_8_ cells

**Figure 5:**
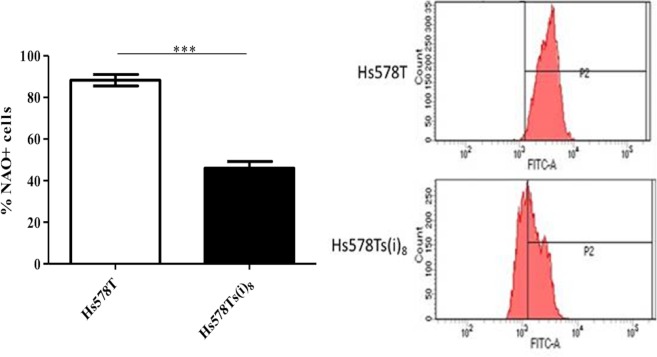
Mitochondria in Hs578Ts(i)_8_ cells show decreased mitochondrial potential compared to that of Hs578T cells. Hs578T and Hs578Ts(i)_8_ cell line variants were stained with 10 µM NAO. The intensity of the NAO staining showed that there is decreased mitochondrial membrane potential in the Hs578Ts(i)_8_ cells compared to the Hs578T cells. Data is expressed as the mean ± SEM of n = 3 experiments, where ***p < 0.001.

We subsequently assessed whether the decrease in oxidative phosphorylation in the Hs578Ts(i)_8_ cell line variant was due to mitochondrial dysfunction, as measured using NAO, a marker of mitochondrial membrane potential. NAO is a lipophilic cation that binds to cardiolipin in the inner mitochondria matrix. When added to cells, NAO accumulates and fluoresces in the inner mitochondrial matrix at high membrane potentials and, therefore, can be used as a measure of mitochondrial membrane potential^21^. As shown in Fig. 5, the reduction in oxidative phosphorylation observed in the Hs578Ts(i)_8_ variant is associated with altered mitochondrial membrane potential, as NAO staining in the Hs578Ts(i)_8_ cell line variant is significantly (p = 0.0006) decreased compared to that in the Hs578T cell line variant. This suggests mitochondrial dysfunction in Hs578Ts(i)_8_ cells.

### Hs578Ts(i)_8_ cells have a significantly increased proportion of cells with CSC phenotype

**Figure 6:**
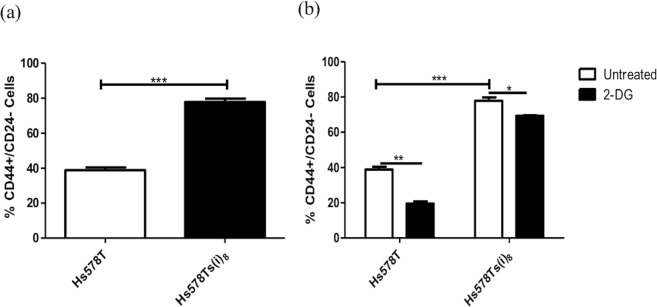
Hs578Ts(i)_8_ cells have an increased CSC population. Analysis of the CSC population showed that (**a**). Hs578Ts(i)_8_ variant, compared to Hs578T, has a significantly increased proportion of CD44^+^/CD24^−^ cells, and (**b**). 2-DG significantly decreased the CSC population in both cell variants. Data is expressed as the mean ± SEM of n = 3 experiments, where *p < 0.05, **p < 0.01, ***p < 0.001.

There is evidence to suggest that tumour growth relies on the cancer CSC population, a cell sub-population which have been shown to be resistant to radiation and chemotherapy. Breast CSC have also been shown to shift from mitochondrial oxidative phosphorylation to fermentative glycolysis, and the proliferation of this CSC population can be inhibited using 2-DG either alone or in conjunction with doxorubicin^22^. Here, analysis of the CSC phenotype showed the Hs578Ts(i)_8_ variant to have a significantly (p = 9.6 × 10^−06^) increased proportion of CD44^+^/CD24^−^ cells as determined by flow cytometry (Fig. 6a). Treatment with 2-DG significantly decreased the proportion of cells with CSC phenotype in both Hs578Ts(i)_8_ (p = 0.01) and Hs578T (p = 0.001) variants (Fig. 6b). This suggests that treatment with 2-DG not only targets glycolysis in TNBC cells, but may also directly affect cells with the CSC phenotype.

## Discussion

In this comparative study, we have successfully shown that 2-DG particularly affects triple negative breast cancer (TNBC) cells that have a very aggressive phenotype. Our results show for the first time that 2-DG can significantly impair migration, invasion and resistance to a form of apoptosis termed *anoikis* of the more aggressive TNBC cell line variant Hs578Ts(i)_8_, with significantly less effects on the parental Hs578T population.

The results presented here indicate that Hs578Ts(i)_8_ cells with the very aggressive phenotype have undergone a metabolic switch, reducing their requirement for oxidative phosphorylation and increasing their rate of glycolysis. Our findings are in agreement with the observations of Otto Warburg termed the Warburg effect. The premise of Warburg’s observations is that cancer cells switch from using their mitochondria to produce ATP energy through oxidative phosphorylation to using glycolysis for ATP production^2^; although not all cancer cells perform this switch - some have levels of oxidative phosphorylation comparable to those of non-malignant cells, while others have altered or non-existent oxidative phosphorylation so this switch cannot be assumed^23^.

Ultimately, the switch towards glycolysis results in increased acid production, which causes normal cell death and enhances degradation of ECM proteins^24–26^; both events facilitate cell migration and invasion, likely enabling metastatic spread. Furthermore, our aggressive cell variant is also more resistant to *anoikis*, the ability of cells to detach from the ECM and other cells and survive in suspension (as would be necessary in the bloodstream), and an essential property to form metastasis in other parts of the body. Since metastasis is the major cause of cancer-associated death, inhibition of glycolysis and subsequent impairment of cell migration and invasion would improve patient outcome.

We hypothesised that the decrease in oxidative phosphorylation in the more aggressive cell line may be due to reduced mitochondrial abundance, reduced substrate supply to the mitochondria and/or dysfunctional mitochondria. From our citrate synthase assays and VDAC1 immunoblots, we established that the mitochondrial biomass is not significantly different between Hs578T and the more aggressive Hs578Ts(i)_8_ cells. We thus tested the effect of substrate supply to the mitochondria using DCA, a known inhibitor of PDK^18^. DCA did not increase OCR in the more aggressive TNBC cells, indicating that decreased substrate supply is most likely not the reason behind the switch to glycolysis. Investigating the mechanism further, we successfully established that mitochondrial membrane potential is significantly decreased in the Hs578Ts(i)_8_ cell variant compared to the parent Hs578T cells. This points to mitochondrial dysfunction as the reason behind the switch to glycolysis in Hs578Ts(i)_8_ cells and also explains their increased sensitivity to 2-DG.

Recent studies have shown that an increased glycolytic phenotype and decreased oxidative phosphorylation are associated with the cancer CSC population^27^, a sub-population of cells within a solid tumour that exhibit CSC characteristics^28^. Furthermore, as previously mentioned, proliferation of purified breast cancer CSCs can be inhibited by 2-DG^22^. Interestingly, we found the proportion of cells with CSC phenotype to be significantly increased in the more aggressive Hs578Ts(i)_8_ cell line variant compared to the Hs578Ts cell population. Similarly to Ciavardelli *et al*.^22^, we found that treatment with 2-DG substantially affected cells with a CSC phenotype decreasing their proportion within the whole cell population. These results show the close association between altered bioenergetics and the increased CSC phenotype in the aggressive population and also suggests a novel way of targeting this cell sub-population, which is known to be resistant to radiation and chemotherapy.

Somewhat unexpectedly, our results showed a more substantial decrease in the CSC population from Hs578T cells than from Hs578Ts(i)_8_ cells. The CSC phenotype is known to show ample plasticity and to be influenced by environmental cues^27^. Furthermore, it has been speculated that several different CSC phenotypes can coexist within tumours, each with unique particular characteristics^29–31^. It could be speculated that, although retaining core characteristics such as CD44/CD24 staining, CSCs from Hs578Ts and Hs578Ts(i)_8_ cells are different and thus show differential sensitivity to 2-DG.

A limited number of studies have suggested that 2-DG may be of benefit as a treatment for breast cancer. Using a TNBC cell line (MDA-MB-231) and an oestrogen-positive breast cancer cell line (T47D), Hadzic *et al*.^32^ were first to report differential susceptibility to 2-DG of human breast cancer cells to normal human cells. Comparing effects of 2-DG and doxorubicin on T47D and a slower growing oestrogen-positive cell line MCF-7, Ahmad *et al*.^33^ reported this treatment to significantly decrease survival of T47D but not MCF-7. Thus, 2-DG may be ideally positioned to target metabolically dysfunctional TNBC cells. Based on the very recent study published by Bizjak *et al*.^34^ where 600 μM of 2-DG -*albeit* in combination with metformin- was found to reduce viability of MDA-MB-231 cells, it could be beneficial for future studies to also investigate dose-responses to lower concentrations of 2-DG prior to progressing to pre-clinical *in vivo* studies.

## Conclusions

Our studies suggest that 2-DG may be beneficial as part of a pharmacological treatment regime for the most aggressive forms of TNBCs which result in the worst outcome from breast cancer. Further expansion of this work to more cell lines and to patients’ specimens, possibly in parallel with evaluating glucose utility by PET as a predictive biomarker for 2-DG use, is now warranted.

## Supplementary information

Supplementary Material

## Data Availability

All data generated or analysed during this study are included in this published data and its supplementary information files.
