# Supplementary material for: 2-Deoxy-D-Glucose inhibits aggressive triple-negative breast cancer cells by targeting glycolysis and the cancer stem cell phenotype
Source: Sci Rep. 2019 Mar 7;9:3788. doi: 10.1038/s41598-019-39789-9 (PMC6405919; doi:10.1038/s41598-019-39789-9)
Supplement: Supplementary file 1 — Supplementary Material [file 41598_2019_39789_MOESM1_ESM.pdf]

Title:

2-Deoxy-D-Glucose inhibits aggressive triple-negative breast cancer cells by targeting glycolysis and the cancer stem cell phenotype

Authors:

Sadhbh O'Neill, Richard K. Porter, Niamh McNamee, Vanesa G. Martinez, Lorraine O'Driscoll

Supplemental Fig. 1. Hs578Ts(i)$_8$ cells, compared to Hs578T cells, have significantly increased migration, invasion and increased resistance to *anoikis.* Compared to Hs578T parent cells, the Hs578Ts(i)$_8$ phenotype is significantly more **(a).** migratory, **(b).** invasive, and **(c).** resistant to *anoikis*. Data is expressed as the mean ± SEM of n=3 experiments, where *p<0.05, **p<0.01, ***p< 0.001.

Supplemental Fig 2. Evaluation of potential alterations in the mitochondrial biomass. Immunoblots on triplicate repeats (P1, P2, P3) of Hs578T Hs578Ts(i)$_8$ cells for VDAC1, normalised to β-actin control, showed no significant differences between Hs578T and Hs578Ts(i)$_8$ cell variants. VDAC1 was probed for, the transmembrane was stripped and re-probed with the anti-β-actin antibody. Due to the large qualities of VDAC1 in the cell lysates, complete stripping did not occur and faint VDAC1 bands are still visible when presenting β-actin. However, their size differences make them clearly distinguishable. In Fig 3. of the manuscript, the immunoblot was cropped to approximately 6 gel bandwidths of the relevant band.

Supplemental Fig 3. DCA does not inhibit growth of Hs578Ts(i)$_8$ cells at 5mM. Treatment of Hs578Ts(i)$_8$ cells with a serial dilution (from 20mM) of DCA showed Hs578Ts(i)$_8$ cell viability unaffected at 5mM.

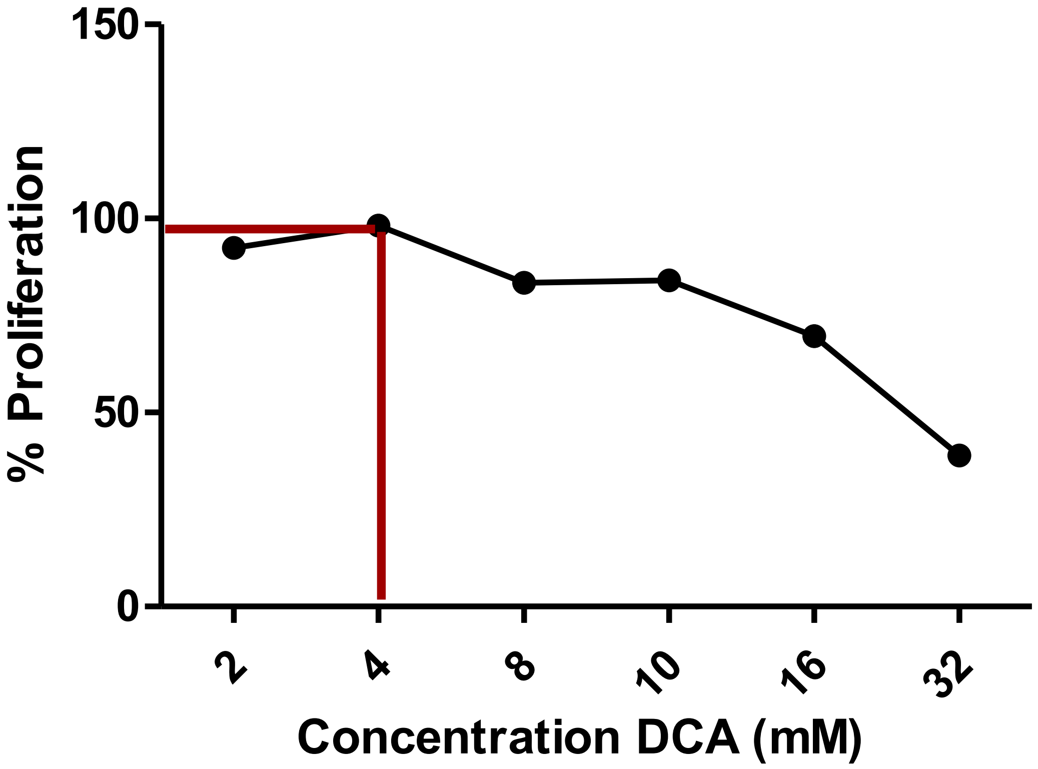

Supplemental Figure 1

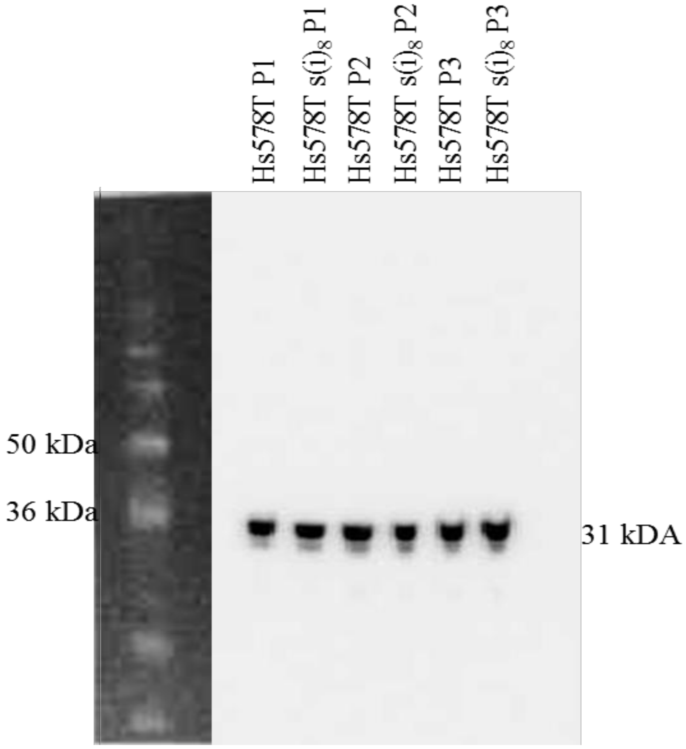

Supplemental Figure 2

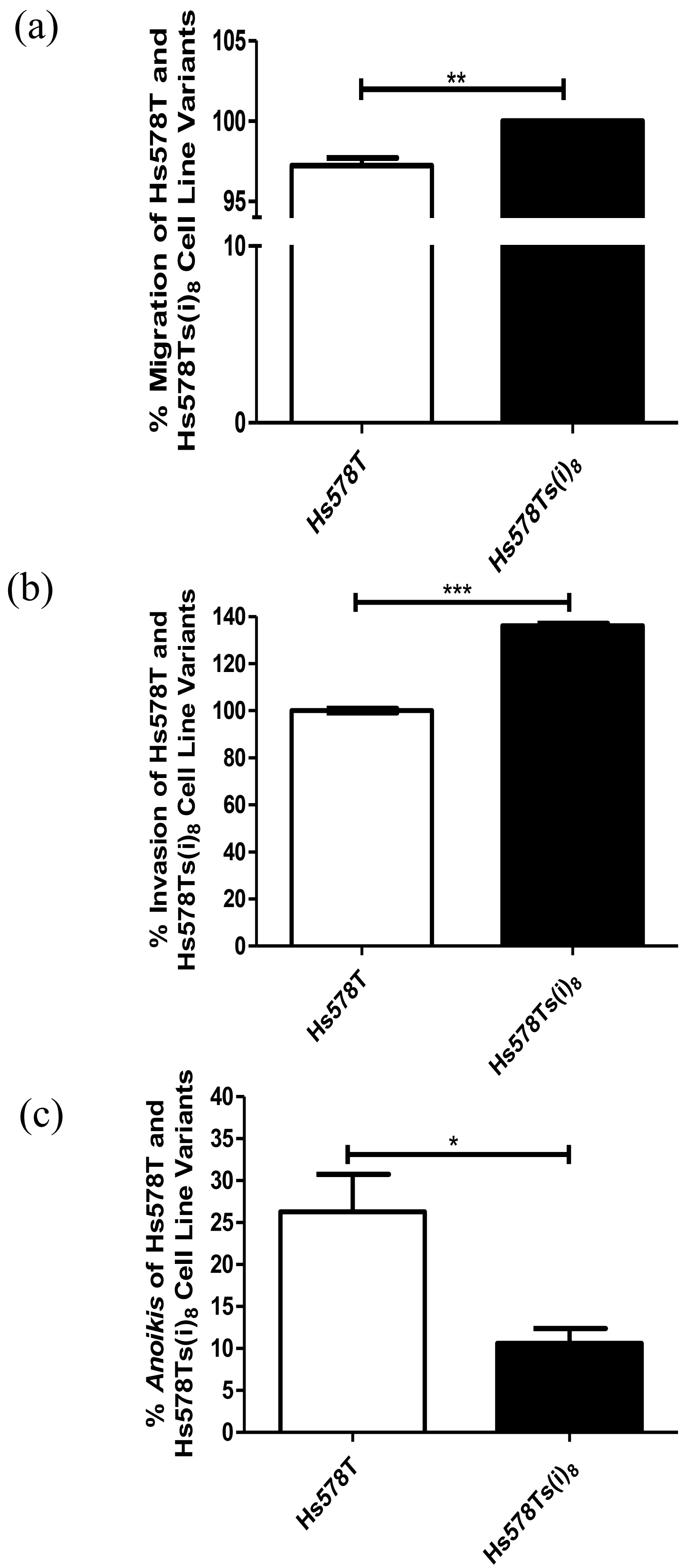

Supplemental Figure 3

Experiments were completed with a lower concentration (600µM) of 2-DG, as well as with 15 mM 2-DG. These experiments showed that 2-DG at 600µM has a significant *albeit* small effect on the migration of Hs578Ts(i)$_8$ cells (see Supplementary Fig. 4). The effect was much less pronounced than that of 15 mM of 2-DG. 2-DG at 600µM had no significant effects on cellular invasion or *anoikis*.

The rationale of using the higher dose of 2-DG is based on our screening work and work by others. We observed that 15 mM was non-toxic to our cells and it is similar to, or lower than, the concentration of 2-DG typical used by other researchers in this field.

For examples, in breast cancer studies:

- in their breast cancer studies Hadzic et al. [Reference 33 in this manuscript] used 20mM 2-DG,
- Ahmad et al. [Reference 34 in this manuscript] used 20mM 2-DG to enhance the susceptibility of breast cancer cells to doxorubicin.
- Many other examples exist including the studies of 2-DG in breast cancer reported by Cheng et al. (2012; Cancer Research 72:2634-2644) where mM concentrations of 2-DG were used.

Furthermore:

- In their Agilent Application Notes "Identifying Metabolic Phenotype Switches in Cancer Cells using the Agilent Seahorse XF Analyser", 100mM 2-DG is used (the cell type was MCF-7 breast cancer cells). *https://www.agilent.com/cs/library/applications/5991-7146EN.pdf*
- When studying the metabolic reprogramming of macrophages: GLUT-1-mediated glucose metabolism drives a pre-inflammatory phenotype, Freemerman et al. used 20mM 2-DG (J. Biol. Chem. 2012; 289(11):7884-7896).
- When studying mitochondrial function of HEK293 cells, Barbi de Moura et al. used 100mM 2-DG. (PLoSOne 2014; 28;9(8):e106028).
- When describing how mTORC dependent metabolic reprogramming is a prerequisite for NK cell effector function, Donnelley et al. used 30mM 2-DG. (J. Immunol. 2014; 193(9):4477-4484).

Thus, 2-DG, when used alone, is typically used at concentrations between **20-100mM**. We used 15mM, which is lower than the range typically used.

Supplemental Fig 4. Effects of 600μM 2-Deoxy-D-glucose on migration, invasion and resistance to *anoikis* of Hs578Ts(i)$_8$ cells. (a). wound-healing assays indicate that 600μM 2-DG significantly decreases the rate of migration of the Hs578T(i)$_8$, although the effect was very limited compared to the effect of 15 mM 2-DG (as shown in Figure 1). **(b).** invasion assays indicate 600μM 2-DG does not significantly decreases the rate of invasion of Hs578T(i)$_8$ cells; **(c).** *anoikis* assays show 600μM 2-DG does not significantly decreases the apoptosis resistance of the Hs578T(i)$_8$ variant. Data is expressed as the mean±SEM of n=3 experiments, where *p<0.05.

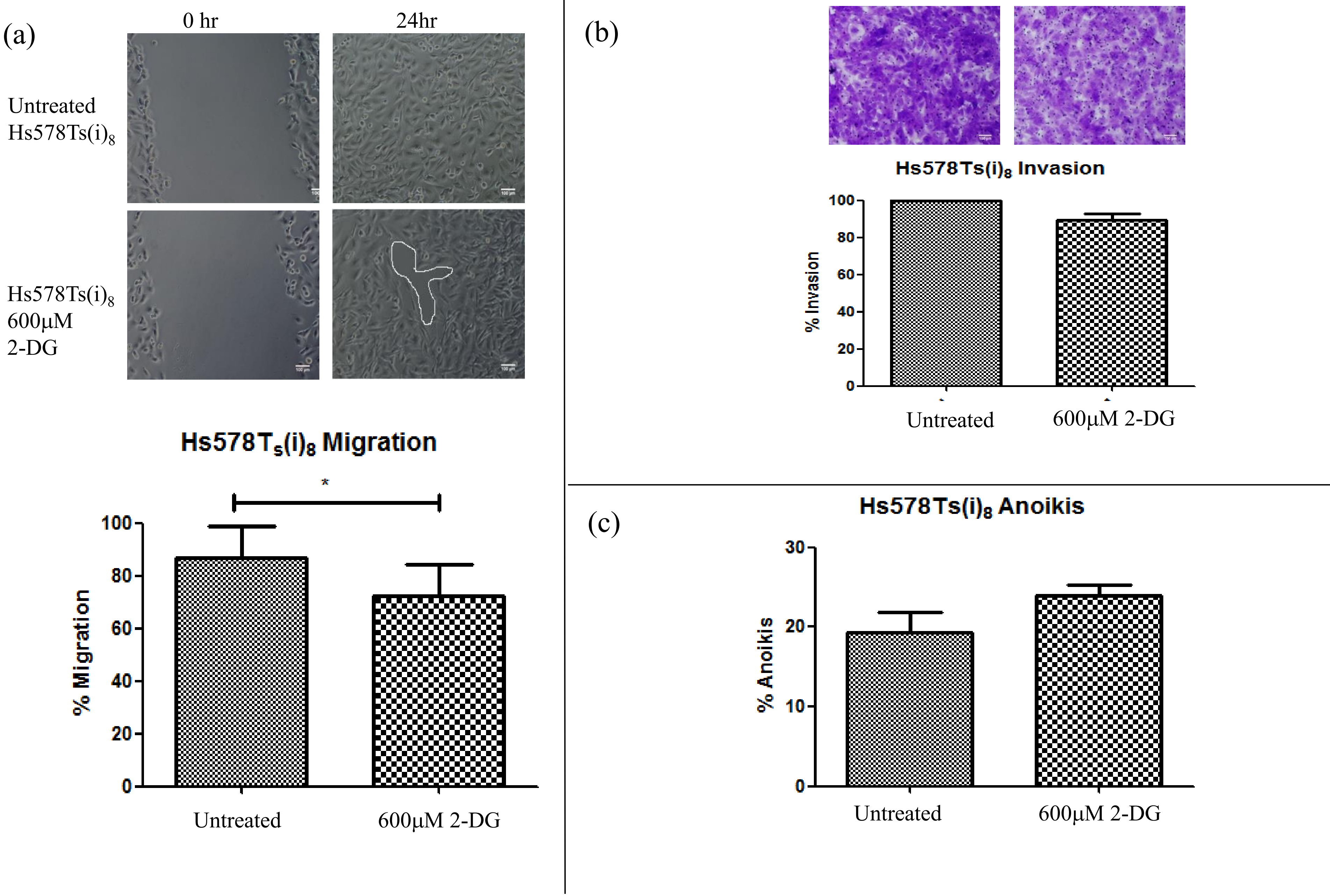

Supplemental Figure 4
